# Jung, Bion and the Crucible of War

**DOI:** 10.1111/1468-5922.13117

**Published:** 2025-07-17

**Authors:** Ann Addison

**Affiliations:** ^1^ London UK

**Keywords:** Bion, Jung, protomental, psychoid, WWI, Bion, Jung, Première Guerre mondiale, psychoïd, proto‐mental, Bion, Jung, Erster Weltkrieg, Psychoid, protomental, Bion, Jung, Prima Guerra Mondiale, psicoide, protomentale, Бион, Юнг, Первая мировая война, психоид, протоментал, Bion, Jung, Primera Guerra Mundial, psicoide, protomental, 比昂, 荣格, 第一次世界大战, 心理物质, 原初思维

## Abstract

WWI had a transformative effect on the lives and ideas of both Carl Jung and Wilfred Bion. Both suffered intense and life‐changing experiences, which they carried with them for the rest of their lives. For Jung, living in neutral Switzerland, the febrile tension of the war emerged in a stream of archetypal imagery, while his daily life interspersed periods of analytic practice and family existence with periods of military service. For Bion, as a tank commander in the British Army, the gritty reality of mud, confusion and shell fire were his daily fare. The impact of the war left its mark on both of them, forging their emotions, their thinking and their theories. In this sense, WWI was a crucible, shaping their experience and their future conceptualizations. This paper reflects on the experiences of both men and on the consequences of these experiences and contemplates the lessons that may be learned from history today.

## Abstract (fr)

La Première Guerre mondiale a eu un effet transformateur sur les vies et les idées de Carl Jung et de Wilfred Bion. Tous deux ont vécu des expériences intenses et bouleversantes, qu'ils ont emportées avec eux pour le reste de leur vie. Pour Jung, vivant en Suisse neutre, la tension fébrile de la guerre émergeait dans une série d'images archétypales, tandis que sa vie quotidienne alternait entre des périodes de pratique analytique et d'existence familiale avec des périodes de service militaire. Pour Bion, en tant que commandant de char dans l'armée britannique, la réalité crue de la boue, de la confusion et des tirs d'obus était son quotidien. L'impact de la guerre a laissé sa marque sur eux deux, forgeant leurs émotions, leur pensée et leurs théories. En ce sens, la Première Guerre mondiale a été un creuset, façonnant leur expérience et leurs conceptualisations futures. Cet article réfléchit sur les expériences de ces deux hommes et sur les conséquences de ces expériences, et envisage les leçons que l'on peut tirer de l'histoire aujourd'hui.

## Abstract (de)

Der Erste Weltkrieg prägte das Leben und die Ideen von Carl Jung und Wilfred Bion grundlegend. Beide erlebten intensive und lebensverändernde Erfahrungen, die sie ihr Leben lang begleiteten. Für Jung, der in der neutralen Schweiz lebte, manifestierte sich die fieberhafte Spannung des Krieges in einer Flut archetypischer Bilder, während sich sein Alltag zwischen Phasen analytischer Praxis und Familienleben und Militärdienstzeiten abwechselte. Für Bion, einem Panzerkommandanten der britischen Armee, war die harte Realität aus Schlamm, Chaos und Artilleriefeuer Alltag. Die Auswirkungen des Krieges prägten beide und schmiedeten ihre Gefühle, ihr Denken und ihre Theorien. In diesem Sinne war der Erste Weltkrieg ein Schmelztiegel, der ihre Erfahrungen und ihre zukünftigen Konzeptuierungen prägte. Dieser Artikel reflektiert die Erfahrungen dieser beiden Männer und ihre Folgen und betrachtet, welche Lehren sich heute aus der Geschichte ziehen lassen.

## Abstract (it)

La Prima Guerra Mondiale ha cambiato le vite e le idee sia di Jung che di Bion. Entrambi hanno accusato gli effetti profondi di quelle esperienze, che li hanno accompagnati per il resto delle loro vite. Per Jung, che viveva nella neutrale Svizzera, la tensione febbrile della guerra emerse con una sequenza di immagini archetipiche, mentre la sua vita quotidiana alternava periodi di lavoro analitico e di vita in famiglia con periodi di servizio militare. Per Bion, in qualità di comandante di un plotone di carri armati nell’esercito britannico, la cruda realtà del fango, della confusione e dei bombardamenti era stato il suo cibo quotidiano. L’impatto della guerra ha lasciato un segno su entrambi, ha plasmato le loro emozioni, il loro pensiero e le loro teorie. In questo senso, la Prima Guerra Mondiale è stata una sfida, e ha modellato le loro esperienze e le future concettualizzazioni. L’articolo riflette sulle esperienze di questi due uomini e sulle conseguenze che queste hanno avuto e si sofferma sulle lezioni che dovremmo imparare dalla storia, ancora oggi.

## Abstract (ru)

Первая мировая война оказала трансформирующее воздействие на жизнь и идеи как Карла Юнга, так и Уилфреда Биона. Оба они испытывали изменившие их жизнь интенсивные переживания, которые пронесли через всю жизнь. У Юнга, жившего в нейтральной Швейцарии, неистовое напряжение войны вылилось в поток архетипических образов, в то время как в его повседневной жизни периоды аналитической практики и семейной жизни чередовались с периодами военной службы. Бион был командиром танка британской армии, и грязь, неразбериха и артиллерийский огонь были суровой реальностью его повседневной жизни. Последствия войны наложили свой отпечаток на них обоих, оказав влияние на их эмоции, мышление и теории. В этом смысле Первая мировая война стала для них суровым испытанием, повлиявшим на их переживания и будущие концепции. В статье анализируется опыт Юнга и Биона и его последствия, а также рассматриваются уроки, которые можно извлечь из истории сегодня.

## Abstract (es)

La Primera Guerra Mundial tuvo un efecto transformador en las vidas e ideas tanto de Carl Jung como de Wilfred Bion. Ambos vivieron experiencias intensas que les cambiaron la vida y continuaron con ellos por el resto de sus vidas. Para Jung, que vivía en la Suiza neutral, la tensión febril de la guerra emergió en un torrente de imágenes arquetípicas, mientras que su vida cotidiana intercalaba periodos de práctica analítica y existencia familiar con periodos de servicio militar. Para Bion, como comandante de tanques del ejército británico, la cruda realidad del barro, la confusión y los bombardeos eran su día a día. El impacto de la guerra dejó huella en ambos, forjando sus emociones, su pensamiento y sus teorías. En este sentido, la Primera Guerra Mundial fue un crisol que moldeó sus experiencias y futuras conceptualizaciones. Este artículo reflexiona sobre las experiencias de estos dos hombres y sobre las consecuencias de las mismas, y contempla las lecciones que en la actualidad pueden extraerse de la historia.

## Abstract (zh)

第一次世界大战对卡尔·荣格和威尔弗雷德·比昂的生活与思想都产生了深远的改变。两人均遭受了剧烈的、改变一生的经历, 这些经历一直伴随着他们的余生。对于居住在瑞士中立国的荣格而言, 战争的紧张情绪以一系列原型意象的形式涌现出来, 而他的日常生活则在分析实践、家庭生活、以及履行军事服务之间交替。对于比昂来说, 作为英军的一名坦克指挥官, 尘土、混乱与炮火的严酷现实则是他的日常。战争的冲击在两人身上都留下了深刻的印记, 塑造了他们的情感、思维以及理论。从这个意义上说, 第一次世界大战就是一个熔炉, 塑造了他们的经历以及他们未来的概念化思想。本文反思了这两位男性人物的经历, 探讨了这些经历的后果, 并思考了从历史中可以汲取哪些教训。

## Introduction

Both Jung and Bion were irrevocably changed by their experiences of WWI. It was a period of turbulence and intense upheaval, which impacted their lives in variety of ways and out of which future growth emerged. For both of them, the Great War served as a crucible, a melting pot providing the ground for an entire raft of future conceptualization as they absorbed and reflected on their own war throughout the remainder of their lives. In this paper, I wish to trace how their personal experiences of the war shaped their ideas, and our reception of them. I wish to examine the nature of those experiences and consider what we may learn from their ensuing thoughts that may be of potential contemporary relevance.

Jung’s visions in *The Black Books* (2020) and his active imagination in his work for *The Red Book* (2009) form a basis for his *psychoid* concept, while Bion’s experiences as a tank commander presage his thinking about groups in the period leading up to, during and after WWII, from which he derived his *protomental* concept.

These two concepts appear to have similar ontological roots. Both relate to a deeply unconscious and unknowable stratum, in which body and mind, self and other are undifferentiated. However, the origins of these concepts lie in radically different experiences, and hence the overarching meaning in each case embraces different questions.

In this paper, I am not taking a political approach. Rather, I am going to situate the history and then infer some of the influences of the war through a literary journey. I shall trace the autobiographical and other related writings of Jung and Bion, in order to elicit their perceptions of the war, their personal responses, and their relationships with those around them.

They each produced a variety of autobiographical accounts, written at different times in their lives and couched in different voices. For Jung, we have *The Black Books* (2020), *Liber Novus* (*The Red Book*) (2009) and his much later written *Memories, Dreams, Reflections* (1963) (*MDR*), as well as significant volumes of his letters (Adler, 1973, 1976; McGuire, 1991). Turning to Bion, we have his *War Memoirs* (1997), *The Long Weekend* (1982), *All My Sins Remembered* (1991) and *A Memoir of the Future* (1990).

In the case of Jung, we post‐Jungians have been led to focus primarily on his myth, his visions, and his profound intra‐psychic experience. Beebe (2014) in an essay on “The Red Book as a Work of Literature” contemplates healing by non‐rational means and considers that:

*The Red Book* provides in literary form a model of how the integrity of the psyche can be restored in the face of cultural processes that threaten to undermine it (Beebe, 2014, p. 109).

Yet Jung also lived a full personal life and saw *this* as an utter necessity to anchor himself in the face of his internal disturbance. And, indeed, in spite of living in a neutral territory, his life was not untouched by the exigencies of war. Less has been written of this aspect of his daily existence but it is nonetheless a significant backdrop to his experience and theorization.

Bion, by contrast, gives us in his diary detailed daily observations of the mud and squalor of his actual war, perhaps seen through the lens of memory and reflection but nonetheless rooted in grim and gritty reality. For him, the relationship with his men was central and heralded the beginnings of his thinking about a collective matrix.

By navigating some of this emotional experience through their memoirs, and by contemplating the surrounding context together with their early conceptualizations, I aim to draw some new comparisons between Jung and Bion.

## The Personal Context

When WWI broke out, Jung was already established in his career, while Bion was still at school.

Jung had a professional reputation based on his researches at the Burghölzli Clinic in Zurich, where he was employed as a psychiatrist, and his collaborations with Freud. He had located the emotional energy and somatic base of unconscious fantasies through his Word Association Tests (WATs) and he had uncovered a mythological layer in the psyche through his observation of inpatients with Dementia Praecox (schizophrenia), enabling him to conclude that emotional experience clusters in unconscious complexes having mythological roots.

While Bion, just 19 when he joined the armed forces on January 4, 1916, described himself as wholly unfledged (1982, p. 104), Vermote notes that the later Bion saw “personality as constituted of layers like the skins of an onion” (Vermote, 2019, p. 214). The different texts that he wrote on his experiences of the war (Bion, 1982, 1990, 1997) give the reader insight into these layers and the fluid way that experience transformed for Bion with the passage of time.

In later life, both men wrote autobiographies. Jung says of *MDR*, “Thus it is that I have now undertaken, in my eighty‐third year, to tell my personal myth” (1963, p. 17). A comparison for Bion may be found in *The Long Weekend* (1982), where he describes his early years, his childhood in India, boarding school in England, and the war. Bion was 85 when he wrote this account. These late life accounts show the accumulation of emotional material refracted throughout life, affecting our reflections. Both these narratives are written in a poetic style, more literary than factual.

## The Experience of WWI

Jung’s war is covered in various texts. His visions and his work on these are set down in *The Black Books* and *The Red Book*, Shamdasani describing the latter as “an experiment that was as much literary as it was psychological and spiritual” (Jung, 2009, p. 98) and Beebe as “a novel in disguise” (2014, p. 113). Jung’s understanding of the meaning of these experiences and his early theoretical conceptualizations out of them follow in other texts, such as “The Transcendent Function” (1916) and “The Role of the Unconscious” (1918a).

Alongside this mythopoeic narrative, we also have Jung’s actual experiences. He lived in a neutral country with all that that meant for Switzerland. He was largely able to continue his analytic practice and his family life; he had an intense association with Maria Moltzer (a Dutch nurse, first a patient and later a Jungian analyst, who worked with Jung as his assistant—Freud implied they had an affair); and he began his relationship with Toni Wolff, which threw him into chaos (Zervas, 2019, p. 185, referring to Protocols, pp. 171–172). He also undertook long periods of military service, during which he treated casualties of war as a medical doctor. Our legacy is his myth, but his life was also grounded in personal reality, and this yielded its own fruits.

In contrast, Bion narrates a terribly real collective experience in *War Memoirs* and in his subsequent writings. He struggled to find meaning in such chaotic and traumatic experience, which dogged him throughout his life, and it is much to our benefit that he wove this trauma into his psychoanalytic thinking.

Rowlands notes of Jung that he “is not just describing the creativity of the psyche, his words also enact and perform it” (2005, p. 2). For him this was a form of healing. Compare this with Angeloch, who says of Bion’s writing, “[t]he form in which the shocking experience is narrated mimetically reproduces the shocks and upheavals, passes them on to the reader as shocks in the act of reading itself” (2021, p. 10). In his case, Bion needed to communicate exactly what the impact of war entailed.

## WWI—Jung and the Melting Pot

*Because I have fallen into the source of chaos, into the primordial beginning, I myself become smelted anew in the connection with the primordial beginning*. (Jung, 2009, p. 179)

This quote from *Mysterium Encounter* seems to encapsulate Jung’s experience of the war. His own accounts of this time focus primarily on his “most difficult experiment”, namely his confrontation with the unconscious. He implies that he was at the mercy of his visions and that they threatened to rend his mind asunder. And yet, he was not free of other tensions, the complexities of his relationships with the women in his life, and his exposure to the consequences of war in Zurich and his military service.

Jung began to be exposed to the horrors of war in his visions (1963, p. 199). He had terrible images of Europe being destroyed by catastrophe. In the Preface to *The Red Book*, Shamdasani (Jung, 2009, p. 18) describes the period from 1912–1914 as being filled with apocalyptic imagery in the arts.

In October 1913, on a train journey to Schaffhausen, Jung had a waking dream of an awe‐ful flood covering the land between the North Sea and the Alps. He saw yellow waves, swimming rubble and the death of countless thousands. The dream was repeated two weeks later, more violently, accompanied by a voice saying, “Look at it carefully, it is completely real, and it will come to pass” (Jung, 2009, p. 124):

I was looking down on the map of Europe in relief. I saw all the northern part, and England sinking down so that the sea came in upon it. It came up to Switzerland, and then I saw that the mountains grew higher and higher to protect Switzerland. I realized that a frightful catastrophe was in progress, towns and people were destroyed, and the wrecks and the dead bodies were tossing about on the water. Then the sea turned to blood. At first, I was only looking on dispassionately, and then the sense of the catastrophe gripped me with tremendous power. (Jung, 2012, p. 44)

Initially, Jung took this to be an internal situation threatening psychosis, the destruction of *his* world. He feared he might go mad.

A dream in January 1914 showed darkness with a reddish glow, a sea of blood foaming over his feet and densely pressed multitudes of the dead flowing past. The internal pressure was intense. Attempting to manage this, he sought to know the living meaning of these visionary experiences:

I was frequently so wrought up that I had to eliminate the emotions. … To the extent that I managed to translate the emotions into images—I was inwardly calmed and reassured. Had I left those images hidden in the emotions, I might have been torn to pieces by them. (Jung, 1963, p. 201)

In July 1914, he was preparing to give a lecture in Aberdeen on schizophrenia and wondered if he was not actually succumbing to one. He kept thinking: “I’ll be speaking of myself! Very likely I’ll go mad after reading this paper” (Jung, 2009, p. 28).

Then, immediately after delivering the lecture, the UK press reported the declaration of war by Austria‐Hungary on Serbia on July 28, 1914, and anticipated the incipient outbreak of war generally. At that point, Jung felt released. He now knew the turmoil was not simply intrapsychic, “not what would happen to him, but to Europe” (Jung, 2009, p. 28).

Jung’s journey home to Switzerland was tortuous. The mobilization of the German army prevented him from travelling directly through France and instead he was obliged to make a detour. First, he travelled to Holland, worried for the safety of Maria Moltzer and intending to accompany her back to Switzerland. Thence, he journeyed through Germany in overfilled trains, standing in corridors, delayed in railway sidings, witnessing troops and already a Zeppelin being shot down (Bennet, 1985, p. 94). Hannah reports that he found the German people “in a peculiar kind of ecstasy”, opening their cellars, giving away food and refusing payment (1999, p. 112). It took him weeks to get home.

The outbreak of war made apparent to Jung the deep subliminal connections between individual fantasies and world events. Shamdasani notes 12 fantasies experienced by Jung in the period between October 1913 and May 1914 that he may have regarded as pre‐cognitive of war: the awe‐ful floods accompanied by the voice saying this is completely real and will come to pass; the mountains rising around Switzerland on the map of Europe and the terrible catastrophe; the killing of the German hero Siegfried; seas of blood, the deaths of thousands, a city annihilated, images of military weapons, human remains and destroyed states (Jung, 2009, p. 29). These may be thought of as early examples of what Jung later came to regard as synchronicity, an internal psychic situation of profound meaning corresponding with an external event. Jung had not yet conceptualized this, but he did envisage the importance of investigating the correspondences between his internal visions and imagery and events in the external world. As Shamdasani writes:

He now conceived of the idea of a work exploring the correspondence between his fantasies and what was taking place in the world, at literal and symbolic levels. This was to become *Liber Novus*. (2012, p. 39)

An important example of such correspondence concerns Jung’s vision of the map of Europe above. Switzerland as a neutral territory did not pass through the war unscathed. The country was surrounded by belligerents, the different language speaking cantons tended to receive news and media in their own language and therefore received differing accounts of the war. Barton (2019, p. 13) writes that the media waged war using public opinion designed to serve the interests of the respective belligerent governments. Borgeaud (1914, p. 872) reported that the differing versions of the news in Switzerland from different national sources, had “the nefarious effect of extending the action of war across their boundaries by encroaching on the battlefield of thought, upon their declared neutrality.” There were doubts about the viability of neutrality.

The Swiss army conscripted men of military age in order to defend their borders against breach and prevent violation of its neutrality. Men, such as Carl Jung, had to spend significant periods engaged in military service, and in his case these periods involved medical work in and, as commandant, management of internment camps hosting prisoners of war transferred from the belligerents. Many of these soldiers were in a shocking state, having amputated limbs, mutilated faces and head wounds, paralysis, and breathing difficulties (Barton, 2019, p. 16). Jung’s work during his periods of military service exposed him to these horrors of war, as well as permitting him periods of relative quiet for study. This experience both collective and personal effectively mirrors the events of his dream of the map of Europe and creates a focus on the voice which told him “it is completely real, and it will come to pass” (Jung, 2009, p. 124).

By taking in and caring for prisoners of war, Switzerland sought to create a cohesive effect uniting its own people and assisting in the engagement with the surrounding belligerents. Internment in Switzerland would prevent soldiers from returning home and making a useful contribution to the war effort. For Switzerland also, in spite of being neutral, war meant rationing and hardship for the Swiss people and a flow of internees ensured an income alleviating in some measure such hardship.

On the one the hand, therefore, Jung was exposed to the actualities of war in his military service. The Introduction to *The Red Book* indicates that in the period from 1915–1917, he spent 67, 34 and 117 days respectively on military service. On the other hand, he continued with his analytic practice and his complex family life in Zurich, which now included Toni Wolff since their relationship started in 1914. The general atmosphere in Zurich was also febrile. Richter (2016, p. 12) refers to “the peculiarly tense and claustrophobic atmosphere of neutral Switzerland in the middle of the Great War” in his account of the beginnings of the Dada movement in Zurich. He quotes Arp:

While the guns rumbled in the distance, we sang, painted, made collages, and wrote poems with all our might. We were seeking an art based on fundamentals, to cure the madness of the age, and a new order of things that would restore the balance between heaven and hell. (Richter, 2016, p. 25)

As Jung wrote to the American psychoanalyst Jelliffe in July 1915:

The sky of Europe becomes more and more dark. We are in a pretty uncomfortable situation in our island. It is interesting to see, how difficult it is even with us, to maintain order against the madness of the people. The madness is most infectious. … One sees no end. More than one million of men are either killed or uncurably wounded, and crippled. (McGuire, 1983, P. 198)

Thus, while war was raging in Europe, and Switzerland, surrounded by warring states, was seeking to navigate the turbulence, Jung was struggling to master his numinous visions. This was a work of mythical proportion. He was also grappling with the turmoil of his personal life against such a backdrop of complex currents. The work he engaged in he conceived as a dialogue with his soul.

In *Liber Novus*, Jung commented, “From the flooding darkness the son of the earth had brought, my soul gave me ancient things that pointed to the future. She gave me three things: The misery of war, the darkness of magic, and the gift of religion” (2009, pp. 375–76). Addison (2017, 2019) describes Jung’s struggles between the spirit of the time, which draws him towards scientific knowledge, and the spirit of the depths, which situates the soul in time immemorial and confers living meaning but which also threatens to overcome him. The spirit of the depths brings his soul into play with the deep primordial beginning, the *unus mundus*, later to be described as the psychoid unconscious. Guided by his soul, *Liber Novus* effectively traces Jung’s individuation process.

The poetic evocation of the individuation process in *Mysterium Encounter* (Maillard, 2014, p. 86) recurs in the series of visions occurring in the short period from end January 1916 to February 8, 1916. These became Jung’s *Seven Sermons to the Dead*, attributed to a Gnostic source. Here, Jung elaborates the notion of a primordial beginning in terms of Pleroma and Creatura. The Pleroma is described as nothingness or fullness, whose qualities are undifferentiated pairs of opposites, such as time and space, good and evil. Creatura on the other hand is confined within time and space; its function is to differentiate or discriminate. The danger for Man is to fall into the Pleroma or undifferentiated state of nothingness. His fight to achieve differentiation leads to the *Principium individuationis*, the process of becoming oneself.

Around the same time, Jung was also drawing his first mandala, which he termed *Systema Mundi Totius* (the structure of the whole world), not at the time understanding its significance. An initial sketch produced in January 1916 showed a central black and yellow annulus surrounded by a band labelled “Pleroma”. (Jaffé, 1979, pp. 75–76). Later, in 1955, Jung wrote:

It portrays the antimonies of the microcosm within the macrocosmic world and its antimonies. (2009, p. 560)

Jeromson (2005/2006, p. 10) indicates that *Systema Mundi Totius* was produced directly after the writing of *The Seven Sermons*, motifs contained in the poetic language of *The Seven Sermons* being echoed geometrically in *Systema Mundi Totius*. The two thus mirror one another as joint symbolic products of Jung’s self‐exploration, leading towards the Self. This is also discussed in *The Art of C. G. Jung*, which reveals that Jung’s handwritten copy of the *Sermones* includes a photograph of *Systema Mundi Totius* (Jung, 2023, p. 116).

Alongside this internal work, Jung was undertaking his various periods of military service. From around January 1 to March 8, 1915, he was on military service in Olten, followed by assignment to invalid transport between March 10 and 12. During these duties, he studied the works of the Gnostics, which led him to his experience of the visions that became his seven sermons.

In 1916, Jung founded the Zurich Psychological Club as an endeavour to bring together members of the analytic community and bring the life of the individual into engagement with the life of the group. His aim was to study the relationship of individuals to the group (Jung, 2020, p. 46). Emma Jung became the first president.

From October to December 1916, Jung served in Herisau. While he was here, he suspended his analytic practice (a letter dated October 22 promises to take a Miss Bowditch into analysis after his return from military service [Adler, 1973]). And, in November 1916, he wrote his early conceptual paper “The Transcendent Function” (1916/1957), attempting to translate his visionary experiences and process for dealing with them into psychological language.

In this paper, Jung makes no mention of his own struggles with his visions, but he describes a process of meaning‐making through the embodied elaboration of unconscious imagery, promoting a union of conscious and unconscious contents. Conscious and unconscious hold complementary positions but such elaboration, through what Jung later termed active imagination, widens the sphere of consciousness as it integrates new unconscious material and leads eventually to a new position indicative of future purpose (the transcendent function). Such amplification *personalizes* the mythological motifs from the unconscious and encourages a purposive and therapeutic unfolding of the Self: “The confrontation of the two positions generates a tension charged with energy and creates a living, third thing” (1916/1957, para. 189).

From June 11 to October 2, 1917, and again in 1918, Jung served as commandant of English military internees at Château d’Oex, of whom there were significant a number. While he was at Chateau d’Oex, Jung daily drew in his military notebook a series of mandalas, 27 in total, each drawn in graphite and carefully dated. Zervas (2018) discusses the progression of these. Jung described them in his “Commentary on *The Secret of the Golden Flower*” as:

A series of European mandala drawings in which something like a plant seed surrounded with membranes is shown floating in the water. Then, from the depths below fire penetrates the seed and makes it grow, causing a great golden flower to unfold from the germinal vesicle. (Jung, 1929, para. 34)

The vesicle becomes a dark central circle, and the seeds scatter. The overall circular symmetry is retained.

On August 5, Jung received a letter from Maria Moltzer that disturbed him. The mandala drawn the next day was broken. He linked this with an internal dialogue he was having about the purpose of his self‐experimentation. He considered this to be nature while a woman (apparently Moltzer) had been asserting that it was art. Elsewhere, he recounts his resistance to this. In Moltzer’s actual letter, she argued that his fantasies should be considered art, and he was upset. He felt the mandala drawing had been destroyed by his emotional state.

The later drawings, however, recovered and became increasingly detailed and developed lotus flower forms until finally these are centred within a cosmic egg. Jung came to see that these mandalas, sometimes broken, were cryptograms representing the state of his Self.

An outbreak of Spanish flu during this period killed a significant number of the internees (Barton, 2019, p. 66). A handwritten letter of December 9, 1918, to an unknown correspondent apologizes for the delay in writing due to “an enormous amount of work” and reports on the health of Ivan Tattersall Hodgkinson referring first to his nervous conditions and his progress towards a more concentrated mental state and then to the “Grippé” or flu that he was now suffering (Jung, 1918b). A footnote in *The Black Books* records that at some point in 1919 Jung contracted the Spanish flu, during which he suffered a very high temperature and feared he might die. Perhaps his ill health was a consequence of nursing such cases. In any event, during his fever, Jung had a series of visions: a turbulent sea, a volcanic island, a soft evening sky, a harbour with boats moored and a tent onshore, all including visions of spheres in the form of circles, each containing a cross, seen as symbols for the Self (Jung, 2023, pp. 131–136). Cambray (2023a, 2023b) notes that his illustrations of these visions capture some essential features of the form of the virus, although this was not currently known, and he links this with the psychoid imagination.

Through this entire period during and after WWI Jung was working on his own individuation and making sense of the madness of the war. *The Red Book* was a work of huge emotional significance, and aspects of it certainly represent psychotic anxiety. His personal life at the start of the war was also in turmoil firstly due to his conflictual relationship with Maria Moltzer and then later due to the tension within his marriage over his relationship with Toni Wolff. This period of his life was marked by his attempts to reconcile the internal with the external, the personal and the collective. The effect of this turbulence within and around him impacted his descriptions of the psyche in so many of his later texts.

In 1917, Jung published a short book, *The Psychology of the Unconscious Processes: An Overview of the Modern Theory and Method of Analytical Psychology* (published in *CW* 7 as “On the Psychology of the Unconscious”). In his preface, dated December 1916, he proclaimed:

The psychological processes, which accompany the present war, above all the incredible brutalization of public opinion, the mutual slanderings, the unprecedented fury of destruction, the monstrous flood of lies, and man’s incapacity to call a halt to the bloody demon—are suited like nothing else to powerfully push in front of the eyes of thinking men the problem of the restlessly slumbering chaotic unconscious under the ordered world of consciousness. This war has pitilessly revealed to civilized man that he is still a barbarian … . But the psychology of the individual corresponds to the psychology of the nation. What the nation does is done also by each individual, and so long as the individual does it, the nation also does it. Only the change in the attitude of the individual is the beginning of the change in the psychology of the nation. The great problems of humanity will never be solved through general laws, but always only through the renewal of the attitude of the individual (1916, p. 4).

Another important early text was “The Role of the Unconscious” published in 1918 (Jung, 1918a), in which Jung grappled with the function of unconscious processes and sought to find meaning in the work he had been doing during the war. He continues his discussion of conscious and unconscious, and comes to the conclusion that:

The union of rational and irrational truth is to be found not so much in art as in the symbol *per se*. … How does a symbol originate? This brings us to the most important function of the unconscious: the *symbol‐creating function* (1918a, paras. 24–25).

[T]he unconscious has a symbol‐creating function only when we are willing to recognize in it a symbolic element. The products of the unconscious are pure nature. … But nature is not, in herself, a guide. … Ships are not guided by the phenomenon of magnetism. We have to make the compass a guide and, in addition, allow for a specific correction, for the needle does not even point to the north. So it is with the guiding function of the unconscious. (1918a, para. 34)

Thus, he concludes that the collective phenomenon that is war must be addressed in the individual:

The individual must consider by what means he can counteract the evil. … In reality only a change in the attitude of the individual can bring about a renewal in the spirit of nations. Everything begins with the individual. (1918a, para. 45)

A series of significant essays deals with the subject of war, directly and indirectly, in *Civilisation in Transition*, *CW* 10. The first, “The Role of the Unconscious”, has just been discussed. Another, “The Fight with the Shadow”, offers an important quote:

I was able to observe how the uprush of dark forces deployed itself in the individual test tube. … There was often terrific suffering and destruction; but when the individual was able to cling to a shred of reason or to preserve the bonds of human relationship … New symbols … appeared, of a collective nature, … reflecting the forces of *order*. There was measure, proportion, and symmetrical arrangement in these symbols, expressed in their peculiar mathematical and geometrical structure. They … are known as mandalas. … [T]hey represent a gleam of hope. (Jung, 1946, para. 450)

In all of this, Jung was studying how the depths of war could be addressed in the individual, and such depths necessarily had to be met in the depths of the unconscious. Here, I think we can see how he began to resort to his conception of the psychoid unconscious as the deepest unknown and irrepresentable layer of the unconscious.

Rowlands observes that all of Jung’s later works “are devoted to finding a form of psychic healing that would avert the acting out of the apocalyptic myth,” and so he experimented with “kinds of writing in which the word has the power to heal” (2005, ix).

## WWI—Bion and the Tanks

Bion was only 19 when he was placed in charge of a tank not long after leaving school, a raw recruit commanding seasoned soldiers. He joined the tanks in Bovington, Dorset, while his mother was staying nearby at Wool. If you visit Bovington Tank Museum in Dorset, England, you can see an exact replica of the model of tank, the Mark IV, that Bion commanded. A notice beside the tank notes, the soldiers who served in her gave her the nickname “mother”. The Mark IV was at the time the most advanced model available (Bion, 1997, p. 12) and came in two types, male and female, differing only in the kinds of guns provided. An armour‐plated fighting vehicle, the tank was capable of a fastest speed of about 4.5 mph, although often movement was barely perceptible and Bion records a day when the tank took 10 hours to complete 4.5 miles (1997, p. 43). Each tank was manned by eight soldiers, namely an officer, a driver and left and right gunners, loaders and gearsmen.

In the claustrophobic confines of one of these early tanks on the front line, Bion witnessed, and himself experienced, shell shock. As he wrote in his diary for September 26, 1917, at Ypres:

All our nerves were in an awful state, and we tried not to think of what was coming. The waiting was awful and seemed to be almost a physical pain—a sort of frightfully “heavy” feeling about one’s limbs and body. (1997, p. 29)

The shelling was one constant roar. The ground was bad, the tanks continually needed to be dug out, their engines would overheat, and the petrol fumes inside the tank would often be very bad (1997, p. 13). There was a lack of wireless communications in WWI; instead each tank carried pigeons for communicating messages between the tanks and HQ (1997, p. 25). This frequently meant that there was confusion as to the latest orders, and uncertainty as to the precise location of the tanks on the map. For Bion, the most important element of this war was his relationship with his men. He was reliant on and responsible for the men in his tank, recounting particularly his driver Lance‐Corporal A. E. Allen and his second in command Sergeant B. O’Toole as good and utterly reliable (1997, p. 8). He worked closely with other officers and tank commanders in his section, writing very personal accounts of his friendship with Quainton with whom he had undertaken his training, and his working relationships with his seconds in command at different stages of the war, Carter, someone absolutely straight and honest (1997, p. 55), and Asser, whose death killed him (1997, p. 203). Then, there are his discussions with Hauser, a fellow officer for whom he had immense and enduring respect (1997, p. 204), in which they reviewed an early form of group dynamics and designed a method for boosting morale amongst the men (1997, p. 89). Every aspect of the war required mutual regard and support and it is clear that Bion was deeply engaged with his fellow men and officers.

After the Ypres Salient, Bion and his company were ordered to a new destination. The tanks were transported by train and driven to Havrincourt, where they engaged with the Germans near Flesquières. At one point, Bion’s tank received a direct hit from a German Howitzer, putting it out of action. The crew evacuated into a nearby communication trench, where Bion reopened fire against considerable odds. For his part in this action of November 20, 1917, Bion was awarded both the British Distinguished Service Order (DSO) and the French Légion d’Honneur (1997, p. viii).

Months of waiting followed. In August 1918, the tanks were trained to a location near Amiens. Bion’s orders were to approach the Amiens‐Roye road and then help the French take Villers‐aux‐Erables. This involved getting all of the tanks across the bridge to Amiens, despite the regular evening shelling. For Bion, “the job rather crushed me. I sat still and felt numb with an almost physical pain” (1997, p. 119). Miraculously, all the tanks made it across the bridge. Walking in front of the tanks, Bion found the strain had a very curious effect:

I felt that all anxiety had become too much; I felt just like a small child that has had a rather tearful day and wants to be put to bed by its mother; I felt curiously like lying down on the side of the road, just as if I was lying peacefully in someone’s arms. (1997, pp. 119–20)

Later that night, the enemy barrage opened, and the fire was so thick that it seemed impossible to continue. At this point, accompanied by a young runner named Sweeting, a shell burst on top of them. Sweeting kept repeating, “‘I’m done for, sir’ … the look in his eyes … the same as that in the eyes of a bird that has been shot” (1997, p. 127). Bion and Hauser were sick and by the end of the battle emotionally and physically exhausted.

All of this is described in Bion’s war diary, written from memory in 1919 soon after the war, at the age of 21, when he had gone up to Oxford. He had not written to his parents during the war, and Chris Mawson comments in the introduction to *War Memoirs* that perhaps the diary was a form of compensation for having found writing to them impossible (1997, p. vii). Parthenope Bion Talamo describes the diary entries as “raw material, with hardly any emotional or intellectual elaboration” (1997, p. 309). They highlight the confusion, the madness, the mud, the danger and the fact that Bion’s military experience was sensual and fragmentary.

Subsequently, Bion returned to this period and especially his memories of Amiens on a number of occasions. In the same volume, there is next a short text entitled “Commentary”, in the form of a dialogue between the 21‐year‐old Bion, tank commander, and the now 75‐year‐old Bion. The young Bion is remembering the fear and loneliness, the feeling of being a mindless robot, never recovering from Amiens. The older Bion was recollecting the rain, the aimless route marches, the mud, the stench of the dead, and the men changed afterwards, become cautious and scared. “I was and am still scared,” he reflects (Bion, 1997, pp. 209–10).

There follows a literary narrative, written in the third person when Bion was 61. It is entitled “Amiens”. It contains memories aroused during a train journey with his wife, Francesca, through an area that held the scene of the death of the young runner Sweeting under Bion’s command. It is cast as a psychological portrait of Captain Bion and his comrades, describing how frightened and panicky he had been when “the shell bursts were incessant; there was no pause between one and another, and it was now impossible to distinguish the sound of any guns—it was lost in one colossal storm of sound” (1997, p. 254). A shell splinter tore out Sweeting’s chest; he asked why he could not cough, calling, “mother, mother” (1982, p. 249). “And then I think he died. Or perhaps it was only me” (1982, p. 249). Later, in *A Memoir of the Future*, Bion writes: “I would not go near the Amiens‐Roye road, for fear I should meet my ghost—I died there” (1991, p. 257).

Another narrative version appears in *The Long Weekend*, written at the end of his life, aged 81. Bion commences the chapter on Ypres, “Wipers, Ypres, the Salient. It had to be held”. The canal had a stain of foreboding on the brightest day. Bion remarked, “The ochreous slime, glistening, featureless, stretching for mile after pock‐marked mile scared me” (1982, p. 126). But “dis‐association, de‐personalization was a way of achieving security,” a personal defence (1982, p. 132). Bion records his guilt at the loss of a number of his men. After all these years, he wrote, “the feelings seemed to have remained and even grown in intensity” (1982, p. 276).

Parthenope Bion Talamo, in a chapter entitled “Aftermath” in *War Memoirs*, writes:

His later theorisation of group dynamics (1948, 1952) made use of the idea of the proto‐mental as the matrix sustaining basic assumption phenomena … I suspect that the experience of panic described in *The diary*, his awareness of the contagious effects of high or low morale, his attempts at a rough sort of “behaviourist” group therapy, … all formed part of the … experience on which his theories lie. (1997, p. 311).

After WW1, Bion studied for a degree in history at Queen’s College, Oxford University, then began his medical career and started a process of working through his experience of the war and a conceptualization of its meaning for him personally. The depths of the horror required deep personal reflection.

In 1942, he was appointed to the War Office Selection Boards (WOSBs) to devise procedures for the selection of potential officers. Here, he developed his leaderless group method, starting from the premise that in war the quality of a soldier’s personal relationships with his fellows is fundamental. Thus, the tension lies between the interests of the group and the soldier’s own interests, and Bion designated this tension as the emotional field to be tested through a real‐life situation. Candidates were placed in a small group and given a practical group task, with no instructions as to how the task was to be carried out. The selection officers observing the group monitored simply how each candidate managed to reconcile his personal ambitions with the requirements of the group and noted what leadership patterns emerged.

Bion took this principle with him to the military psychiatric Northfield Hospital, to which he was transferred in 1943, joining Rickman there. Together, they set about adapting the leaderless group method to the training wing in the hospital.

Bion wrote two papers covering these experiments, “The Leaderless Group Project” and “Intra‐Group Tensions in Therapy: Their Study as the Task of the Group”, the latter constituting the first chapter of *Experiences in Groups*. The first paper shows Bion beginning to view the hospital as if it were a combat unit. The later paper takes this analogy a step further. He writes of the *theatre of war*, announcing that in the chaos of the wards, he became convinced that what was necessary was:

[T]he sort of discipline achieved … by an experienced officer in command of a rather scallywag battalion.... [W]hat sort of discipline is that? In face of the urgent need for action I sought, and found, a working hypothesis. It was, that the discipline required depends on two main factors: (i) the presence of the enemy, who provided a common danger and a common aim; and (ii) the presence of an officer who … respects the integrity of his men, and is not afraid of either their goodwill or their hostility. (Bion & Rickman, 1943, pp. 12–13)

These men were not to be thought of as cannon‐fodder to be returned to their units as fast as possible; instead, the common enemy was to be seen as “the existence of neurosis as a disability of the community”. He said:

Anyone with a knowledge of good fighting regiments in a theatre of war would have been struck by certain similarities in outlook in the men of such a unit and the men of the training wing. (1943, p. 22).

At this point, Bion was starting to contemplate the notion of a container: “I found it helpful to visualize the projected organization of the training wing as if it were a framework enclosed within transparent walls. Into this space the patient would be admitted at one point” (1943, pp. 14–15). The hospital was treated as a social field, and individual and community were viewed as equally important interacting elements.

This was the beginning of Bion’s group work. Still referring to “war”, he gradually distances himself from similarities with the conditions of war, first in generalized comparisons:

The point that I wish to make is that the group is essential to the fulfilment of a man’s mental life—quite as essential to that as it is to the more obvious activities of economics and war. (1961, p. 54)

Bion began to talk about a group mentality, in which material hostile to the purposes of the group, a negative unacknowledged system, would be disavowed. In the group, whatever it may appear on the surface, the situation is charged with emotions which exert a powerful, and frequently unobserved, influence on the individual. As a result, his emotions are stirred to the detriment of his judgment (1961, pp. 39–40).

This innovative concept led eventually to the notion of the work group and the three basic assumption (ba) groupings: fight‐flight (baF), pairing (baP) and dependency (baD). Describing this model, he reflects indirectly on its application to war:

The glaring difficulty is to state what basic assumption is operative in a large group; for example, are we to say that the ba in a nation at war is baF? And if so, is it true that this would hold for all parts of the nation—for example, the agricultural community? If we assume that a nation at war exemplifies baF, are we to assume that the nation in question provides an intelligible field of study for the phenomena associated with that basic assumption? (1961, p. 128)

Then, he arrives at an internalized view, where pressure to progress leads to schism.

When the dependent group or the fight‐flight group is active, a struggle takes place to suppress [a] new idea because it is felt that the emergence of the new idea threatens the status quo. In war, the new idea—be it a tank or a new method for selecting officers—is felt to be “new‐fangled”, i.e. opposed to the military bible. (1961, p. 155)

The schismatic group attempts to solve its problem by internal as opposed to external war. Finally, he proposes that:

The individual is a group animal at war, both with the group and with those aspects of his personality that constitute his “groupishness”. (1961, p. 168)

The interesting dynamic in this chronological development is the way that Bion, from the outset in WWI to his developing theory of groups associated with WWII and afterwards, contemplates the relationship of the individual to the group, and gradually shifts the conflictual terrain internally, converting the reality of war to a metaphorical war that is both external and internal, thereafter arriving at his protomental theory. Another interesting area is his attitude toward memory, his memories of the war, the lenses through which he sees war, literally and with rawness in the first instance, then metaphorically in his theoretical conceptualizations, and finally with a polyphonic poetry in his autobiographical writings.

## Conclusion

Both Jung and Bion made full use of their war experience in their later theorizations, at the same time producing profound literary products.

Jung’s writings are individual, visionary, a *tour de force* of the unconscious, the emotional grip and intense pressure of its energy, the sheer necessity of addressing it for the sake of psychic survival in the face of psychotic anxiety. While his actual experience confronted him with the reality of war, his intra‐psychic experience was mythological and led him to reflect deeply on the nature of God, the interface of the individual with the collective, and the relationship of the psyche to external reality. It is no surprise that his psychoid concept should be conceived in terms of the unknowable and the numinous in an attempt to embrace the total situation and in some way compensate for the madness of WWI. Even though the lens through which we view his work magnifies the visionary more than the personal, we should not lose sight of the fact that this is not Jung’s whole story. As well as seeing the casualties of war, his personal life was also turbulent; it involved engagement with conflict, emotionally in his relationships, with the women in his life, and with Freud and other associates. All of this is to be found in his autobiographical writings and in his later conceptualizations.

Bion’s writings depict the visceral horror of life on the front line, in the trenches, and in the noxious cabin of his tank. His war was about fragmentation, chaos, squalor and literal survival. For him the relationship with his men was the matrix of life. The lens of memory does not soften the pain but shows his ongoing attempt to assuage his guilt over those who were lost and to make sense of the senselessness of the war. This led him also to deep reflection on the relationship of the individual to the group, and on the intra‐ and inter‐psychic “war” that this entails. His protomental concept reflects these dynamics, the behaviours of groups and their links with the psychobiological realm.

It is very clear that WWI affected both Jung and Bion profoundly, and had a long reach, significantly impacting, shaping, and driving their later conceptualizations. I have only been able to hint here at this legacy.
